# The Development of Persistent Gastrointestinal Symptoms in Patients With Melanoma Who Have Had an Immune Checkpoint Inhibitor-Related Gastrointestinal Toxicity

**DOI:** 10.14309/ctg.0000000000000746

**Published:** 2024-07-12

**Authors:** Sanskriti Varma, Keri Sullivan, Jamie DiCarlo, Alexandra Coromilas, Kyle Staller, Michael Dougan

**Affiliations:** 1 Division of Gastroenterology, Massachusetts General Hospital, Boston, Massachusetts, USA;; 2 Division of Gastroenterology, Massachusetts General Hospital, Center for Neurointestinal Health, Boston, Massachusetts, USA;; 3 Harvard Medical School, Boston, Massachusetts, USA;; 4 Department of Dermatology, NewYork-Presbyterian Hospital, Columbia University Irving Medical Center, New York City, New York, USA.

**Keywords:** immune checkpoint inhibitor, disorders of gut brain interaction, inflammation

## Abstract

**INTRODUCTION:**

Immune-related adverse events (irAE) secondary to immune checkpoint inhibitors (ICI) have gastrointestinal (GI) manifestations, including gastritis, enteritis, and/or colitis. The long-term sequelae of ICI-associated GI toxicities (GI-irAE), particularly the development of disorders of gut-brain interaction, are not well known. We characterized the incidence of persistent GI symptoms after GI-irAE.

**METHODS:**

This is a retrospective study of adults with melanoma treated with ICI and diagnosed with GI-irAE at our institution from 2013 to 2021. All patients had endoscopic and histologic evidence of GI-irAE. The primary outcome was incidence of persistent GI symptoms (diarrhea, abdominal pain, bloating, constipation, fecal incontinence, nausea, vomiting) after resolution of GI-irAE. Hazard ratios evaluated the association between parameters and time to persistent GI symptoms.

**RESULTS:**

One hundred four patients with melanoma (90% stage IV disease) and GI-irAE met inclusion criteria. Thirty-four percent received anti-cytotoxic T lymphocyte-associated protein-4 therapy, 33% anti-programmed death-1, and 34% dual therapy. Patients were treated for GI-irAE for an average of 9 ± 6 weeks. Twenty-eight (27%) patients developed persistent GI symptoms 1.6 ± 0.8 years after GI-irAE. The most common symptom was constipation (17%), followed by bloating (8%) and diarrhea (5%). Over 453 person-years, the incident rate was 6.2% per 100 person-years. Use of cytotoxic T lymphocyte-associated protein-4 single or dual therapy was associated with a 3.51× risk of persistent GI symptoms (95% confidence interval 1.20–10.23).

**DISCUSSION:**

In this cohort of melanoma patients who experienced GI-irAE, 26% developed persistent GI symptoms, most frequently constipation. Future studies should characterize the GI sequelae after GI-irAE, which may shed light on disorders of gut-brain interaction pathogenesis and improve the lives of cancer survivors.

## INTRODUCTION

Immune checkpoint inhibitor (ICI) therapy has revolutionized the treatment of solid and hematologic malignancies and has improved the outcomes of patients with advanced melanoma. The 5-year overall survival rate for patients with metastatic melanoma has risen to about 50% (1). ICIs are monoclonal antibodies that primarily target 2 immune regulatory pathways: cytotoxic T lymphocyte-associated protein (CTLA)-4, programmed death (PD)-1 or its ligand, and lymphocyte activation gene-3 (2). ICIs block regulatory receptors that inhibit effector T-cell activation. Therapeutically, blockade of these receptors leads to augmented responses against tumor-expressed targets, but inhibiting these receptors leads to loss of tolerance to self-proteins and commensal bacteria (3–5). This produces a wide variety of inflammatory toxicities, collectively referred to as immune-related adverse events (irAE).

The most significant effect of ICI therapy tends to be on the barrier organs, including the gastrointestinal (GI) tract (5,6). CTLA-4 inhibitors alone or in combination regimens have the greatest risk for developing irAE (7,8). GI-irAE may occur in up to 40% of these patients (9,10). While ICI colitis is the most frequently reported GI-irAE, these phenomena can occur throughout the luminal GI tract, and 1 study suggests that the most common site of mucosal inflammation is the stomach, based on pathology assessment (11).

Disorders of gut-brain axis (DGBI), including irritable bowel syndrome (IBS) and functional dyspepsia, are highly prevalent conditions with bothersome GI symptoms in the absence of structural abnormalities. Our understanding of the pathophysiology of these conditions has evolved significantly in the recent decades, although remains limited. Inflammatory processes may be 1 component, and the link between the enteric nervous and immune systems is becoming increasingly recognized. For example, studies have shown that mucosal infiltration of immune cells may result in increased release of nociceptive mediators that activate sensitized neurons, leading to visceral hypersensitivity (12,13). Mast cell dysfunction may also disrupt epithelial barriers, alter mucosal permeability, and lead to changes in bowel function and pain perception (14).

Meta-analyses have shown an increased risk of IBS in patients who experienced an episode of acute infectious gastroenteritis, with an incidence up to 35%, further supporting this postinflammatory mechanism of DGBI (15). While inflammatory bowel disease (IBD) is primarily thought of as an immune-mediated disease, up to one-third of patients with IBD will receive a concomitant diagnosis of IBS, even after achieving deep remission (16,17). There are however limited data on the development of DGBI or persistent GI symptoms in patients who have had an inflammatory toxicity in the GI tract from ICI therapy. To our knowledge, this is the first study to characterize the incidence and distribution of persistent GI symptoms after GI-irAE. We believe that this is an initial step in better understanding the development of postinflammatory DGBI.

## METHODS

**Figure 1.**
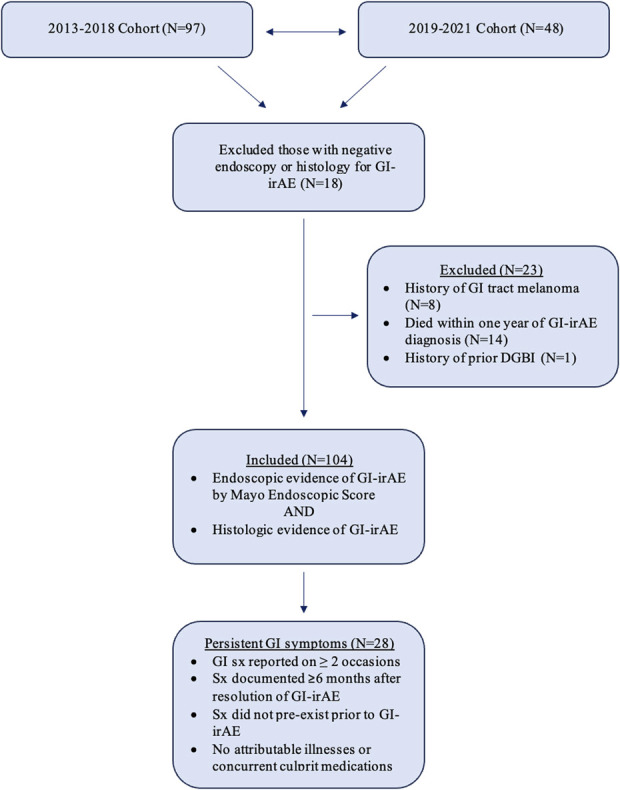
Patient population. Cohort = patients with melanoma aged at least 18 years with prior immune checkpoint inhibitor exposure who have GI symptoms and underwent standard-of-care endoscopy either managed at a subspecialty clinic or identified by the Research Practice Data Registry. DGBI, disorders of gut-brain interaction; GI, gastrointestinal; GI-irAE, gastrointestinal immune-related adverse event; Sx, symptoms.

This is a single-center retrospective cohort study of patients with melanoma at Massachusetts General Hospital, a quaternary care referral center, who received treatment with a CTLA-4 and/or PD-1 inhibitor at any point between 2013 and 2021. Adult patients (≥18 years) with melanoma were included if they experienced a GI-irAE (gastritis, enteritis, and/or colitis), the primary exposure. Cases between 2013 and 2018 were identified based on a well-curated database of patients who were seen at a subspecialty clinic at our institution with the above criteria. Cases between 2019 and 2021 were identified using the Research Practice Data Registry, a data warehouse of all inpatient and outpatient visits within the Mass General Brigham Network through the use of diagnostic *International Classification of Diseases* *Ninth Revision* (*ICD-9*) and *ICD*, *Tenth Revision* (*ICD-10*) codes for colitis and gastroenteritis (K52.x), followed by protocolized chart review (18). Each case was reviewed to confirm a diagnosis of GI-irAE based on endoscopic and histologic evidence. Endoscopic evidence was graded using the Mayo Endoscopic Score, which includes features of erythema, changes in vascular pattern, friability, bleeding, erosions, and/or ulcers. Histologic evidence included active inflammation with neutrophilic crypt microabscesses, prominent crypt epithelial apoptosis, crypt atrophy/dropout or distortion, increased intraepithelial lymphocytes, surface epithelial injury, paneth cell metaplasia, and/or basal lymphoplasmacytosis (19). We excluded patients with a preexisting history of any Rome IV DGBI (as determined by chart review of physician notes and *ICD-9* and *ICD-10* codes present in the medical record; e.g., functional dyspepsia, chronic nausea vomiting syndrome, cyclic vomiting syndrome, IBS, functional constipation/chronic idiopathic constipation, functional diarrhea, functional abdominal bloating/distension, unspecified functional bowel disorder, centrally mediated abdominal pain syndrome, fecal incontinence, globus) before GI-irAE, those who died within 1 year of GI-irAE, and those with GI tract melanoma. This study was approved by the Institutional Review Board at Massachusetts General Hospital (Figure 1).

The primary outcome was incidence of persistent GI symptoms, including at least one of the following symptoms: nausea, vomiting, abdominal pain, bloating, constipation, diarrhea, and/or fecal incontinence. Patients were considered to have developed persistent GI symptoms if they reported any of the above to their physician on ≥2 occasions, documented at least 6 months after the resolution of GI-irAE (determined by a combination of repeat endoscopy, laboratory results, and/or clinical features), and if the symptom did not preexist before the GI-irAE. We examined the medical record to ensure that these symptoms were not attributable to other illnesses or concurrent medications that could account for their GI symptoms. Physician notes, laboratory results, and diagnostic workup at the time of visit for the persistent GI symptom were manually reviewed. If patients were on opiates, ICI therapy, or chemotherapy at the time of their physician visit for persistent GI symptoms, then they were not considered to have developed GI symptoms attributable to GI-irAE.

GI-irAE symptom severity was defined using the Common Terminology Criteria for Adverse Events (CTCAE) version 5.0 for nausea, vomiting, and diarrhea. Symptoms were graded as mild (CTCAE 1), moderate (CTCAE 2), and severe (CTCAE 3–4). Endoscopy reports were manually reviewed, and endoscopic severity was defined by the performing endoscopist using the Mayo Endoscopic Score. The Mayo Endoscopic Score is part of a clinical system devised at Mayo Clinic, Rochester, Minnesota, that is used to quantify the degree of inflammation in the GI tract for patients with ulcerative colitis and GI-irAE (20).

Information was extracted regarding ages at the time of melanoma and GI-irAE diagnoses and sex. We obtained data regarding malignancy history, including melanoma stage (I–IV), immunotherapy type (CTLA-4, PD-1, combination regimen) and duration, and concurrent cancer therapies (neoadjuvant therapy). Variables regarding GI-irAE treatment were collected, including history of hospitalization for treatment/diagnosis, history of biologic use, type and number of biologic doses used, steroid use, duration of treatment, and time to resolution of symptoms.

Statistical analysis was performed using Stata 15.2 (StataCorp, College Station, TX). Continuous variables were summarized using means and standard deviations and compared using the Student *t* test. Categorical variables were presented as proportions and compared using the χ^2^ test. Cox proportional hazards modeling was used to test the associations between parameters and time to persistent GI symptoms. The multivariable model included variables with an association (*P* < 0.1) on univariate analyses. Associations were considered statistically significant at a *P* value <0.05.

## RESULTS

We identified a total of 104 patients with melanoma (90% with stage IV disease) and history of GI-irAE that met inclusion criteria (Table 1). The mean age at the time of melanoma diagnosis was 57 ± 14 years and at the time of GI-irAE was 60 ± 14 years, and most of the population was male (46%). Thirty-four percent received anti-CTLA-4 therapy, 33% anti-PD-1, and 34% dual therapy with anti-CTLA-4 and anti-PD-1. The mean duration of ICI therapy was similar between those who did and did not develop persistent GI symptoms (9 ± 6 months, *P* = 0.71). Ninety-two patients (88%) had a primary diagnosis of ICI colitis, followed by enteritis (n = 25, 24%), gastritis (n = 22, 21%), and esophagitis (n = 3, 3%). The majority with GI-irAE had mild symptoms (49%) and mild Mayo endoscopic severity (54%) (Table 2). Patients were treated for GI-irAE for an average of 9 ± 6 weeks: the majority received steroids (94%) and half (50%) received biologics, a distribution of treatment similar to that seen in our prior studies (21). The time to symptom resolution was similar between both groups (5 ± 5 months, *P* = 0.17).

**Table 1.** Melanoma characteristics of patients who have had a GI-irAE dichotomized by the presence of persistent GI symptoms (N = 104)

	All	(+) Persistent GI symptoms (N = 28)	(−) Persistent GI symptoms (N = 76)	*P* value
Age at time of melanoma diagnosis, yr	57 (14)	57 (14)	57 (14)	1.0
Age at time of GI-irAE, yr	60 (14)	59 (14)	60 (14)	0.78
Sex (female)	46 (44%)	13 (46%)	33 (43%)	0.78
Melanoma stage				0.60
I	3 (3%)	0 (0%)	3 (4%)	
II	1 (1%)	0 (0%)	1 (1%)	
III	10 (10%)	2 (7%)	8 (11%)	
IV	90 (87%)	26 (93%)	64 (84%)	
ICI therapy type				
CTLA-4 inhibitor (single agent)	35 (34%)	24 (86%)	56 (61%)	0.02
PD-1 inhibitor (single agent)	34 (33%)	14 (50%)	55 (72%)	0.03
Dual therapy (CTLA-4 and PD-1 inhibitors)	35 (34%)	10 (36%)	25 (33%)	0.79
ICI therapy duration, mo	9 (6)	9 (5)	9 (6)	0.71

Values are shown as N (%) for proportions and mean (SD) for continuous variables. *P* values compare those with and without persistent GI symptoms.

CTLA, cytotoxic T lymphocyte-associated protein; GI, gastrointestinal; ICI, immune checkpoint inhibitors; irAE, immune-related adverse events; PD, programmed death.

**Table 2.** GI characteristics of patients who have had a GI-irAE dichotomized by the presence of persistent GI symptoms (N = 104)

	All	(+) Persistent GI symptoms (N = 28)	(−) Persistent GI symptoms (N = 76)	*P* value
GI-irAE subtype				
Colitis	92 (88%)	24 (86%)	68 (89%)	0.60
Enteritis	25 (24%)	7 (25%)	18 (24%)	0.89
Gastritis	22 (21%)	9 (32%)	13 (17%)	0.10
Esophagitis	3 (3%)	1 (4%)	2 (3%)	0.80
CTCAE severity				0.85
Mild	51 (49%)	13 (46%)	38 (50%)	
Moderate	40 (38%)	12 (43%)	28 (37%)	
Severe	13 (13%)	3 (11%)	10 (13%)	
Endoscopic severity				0.82
Mild	56 (54%)	15 (54%)	41 (54%)	
Moderate	36 (35%)	9 (32%)	27 (36%)	
Severe	12 (12%)	4 (14%)	8 (11%)	
Hospitalization for GI-irAE	36 (35%)	11 (39%)	25 (31%)	0.44
Biologic use				
None	52 (50%)	18 (64%)	34 (45%)	0.07
Anti-TNFα	49 (47%)	9 (32%)	40 (53%)	0.06
Anti-integrin	6 (6%)	1 (4%)	5 (7%)	0.56
Systemic glucocorticoids	98 (94%)	27 (96%)	71 (93%)	0.56
Treatment duration, wk	9 (6)	8 (5)	10 (6)	0.20
Time to symptom resolution, wk	5 (5)	4 (5)	5 (5)	0.17
Persistent GI symptoms				
Nausea	—	3 (3%)	—	
Vomiting	—	0 (0%)	—	
Abdominal pain	—	0 (0%)	—	
Bloating	—	8 (8%)	—	
Diarrhea	—	5 (5%)	—	
Fecal incontinence	—	1 (0.1%)	—	
Constipation	—	18 (17%)	—	
Time to development of persistent GI symptoms, yr	—	1.6 (0.8)	—	
History of psychiatric comorbidity				
Anxiety	7 (7%)	2 (7%)	5 (7%)	0.92
Depression	11 (11%)	5 (18%)	6 (8%)	0.14
Other	3 (3%)	0 (0%)	3 (4%)	0.29

Values are shown as N (%) for proportions and mean (SD) for continuous variables. *P* values compare those with and without persistent GI symptoms. The time to development of functional GI symptoms (years) is defined as the time between date of GI-irAE diagnosis and date of first clinic visit at which GI symptom was documented.

CTCAE, Common Terminology Criteria for Adverse Events; GI, gastrointestinal; irAE, immune-related adverse events; TNFα, tumor necrosis factor alpha.

Twenty-eight (27%) patients developed persistent GI symptoms at a mean of about 1.6 ± 0.8 years after GI-irAE (Table 2). The most common symptom was constipation (17%), followed by bloating (8%) and diarrhea (5%). During a total of 453 person-years of follow-up, the incident rate for persistent GI symptoms was 6.2% per 100 person-years.

There were no differences in the age, sex, or melanoma stage between those who did and did not develop persistent GI symptoms. A greater proportion of patients who developed persistent GI symptoms underwent treatment with a CTLA-4 inhibitor (86% vs 61%, *P* = 0.02), while similar proportions underwent therapy with dual CTLA-4 and PD-1 inhibitors (36% vs 33%, *P* = 0.79). There were no major differences between the rates of mild, moderate, or severe symptoms or endoscopic findings. Notably, there was a trend toward decreased development of persistent GI symptoms among patients who received anti-tumor necrosis factor alpha (TNFα) therapy (53% vs 32%, *P* = 0.06).

In a multivariable, Cox proportional hazards model adjusting for age and sex, use of CTLA-4 single or dual therapy was associated with a 3.51× increased risk of persistent GI symptoms (95% confidence interval 1.20–10.23, Table 3). As GI-irAEs were not uniformly treated with biologics, we also performed a subgroup analysis of the multivariable Cox proportional hazards model, stratified by biologic exposure. CTLA-4 therapy was not statistically associated with an increased risk of persistent GI symptoms in either those exposed to biologics or in those who were biologic naïve, although persistent GI symptoms were more common in those who were biologic naïve (hazard ratio 3.09, 95% confidence interval 0.88–10.82, *P* = 0.07).

**Table 3.** Cox proportional hazards model for time to persistent GI symptoms among patients with history of GI-irAE (N = 104)

	Age- and sex-adjusted model (HR, 95% CI)	Full model (HR, 95% CI)
Age at time of GI-irAE, per 10 yr	0.99 (0.75–1.29)	0.94 (0.71–1.25)
Sex		
Male	Reference	Reference
Female	1.23 (0.56–2.68)	1.38 (0.61–3.08)
Treatment		
PD-1 inhibitor single therapy	—	Reference
CTLA-4 single or dual therapy	—	3.51 (1.20–10.23)

Full model adjusts for age, sex, CTLA-4 inhibitor, and PD-1 inhibitor use. Reduced model adjusts for age and sex.

CI, confidence interval; CTLA, cytotoxic T lymphocyte-associated protein; GI, gastrointestinal; HR, hazard ratio; irAE, immune-related adverse events; PD, programmed death.

## DISCUSSION

In this retrospective study of patients with melanoma who underwent ICI therapy and developed a GI-irAE, we found that 27% of patients developed persistent GI symptoms, with constipation being the most common. Undergoing therapy with CTLA-4 inhibitors (single or dual) relative to PD-1 single therapy was associated with greater risk for developing persistent GI symptoms. Neither symptom severity nor endoscopic severity was associated with the development of persistent GI symptoms, although absence of association should be interpreted through the lens of the sample size. Our findings suggest the need for vigilance to postinflammatory GI symptoms in cancer survivors previously treated with immunotherapy and add to a growing body of evidence indicating long-term physiologic changes induced by ICI therapy (22). In addition, these findings provide further evidence-linking activation of intestinal immune cells to modulation in the function of the enteric nervous system and provide a potential new model for directly studying the mechanisms underlying this interaction, similar to what has already been done using these drugs to probe basic mechanisms of mucosal tolerance (23,24).

The enteric nervous system senses and reacts to the dynamic ecosystem of the GI tract by translating chemical cues and protein mediators from the environment into neural impulses that propagate throughout the gut and the central nervous system (25,26). Tissue specimens from the GI tract of patients affected by GI dysmotility disorder show dense lymphocytic and plasma cell infiltrate predominantly confined to the myenteric plexus and submucosal ganglia. These changes have been associated with progressive neuronal degeneration and sometimes complete loss of enteric neurons (27,28). Experiments characterizing this infiltrate have shown that most of these cells express either CD4 or CD8 T cells in an approximate 1:1 ratio (29,30). While the underlying cellular basis of persistent GI symptoms is beyond the scope of our current study, the findings do support that inflammatory insults, such as GI-irAE from ICI, may contribute to changes in enteric nerve function; this system provides an elegant and tractable means for studying the mechanisms underlying these changes.

We found that those with a history of CTLA-4 use (as either single or combination therapy) had a greater risk for developing persistent GI symptoms, independent of the apparent severity of mucosal inflammation, suggesting a distinct role for CTLA-4 in the mechanism. CTLA-4 is a decoy receptor for the T-cell costimulatory proteins B7-1 and B7-2, preventing interaction between these costimulatory proteins and the receptor CD28, and ultimately prohibiting activated dendritic cells from further stimulating T-cell activation (23). By contrast, PD-1 sends a direct inhibitory signal through associations with phosphatases and is predominantly expressed on T cells after repeated stimulation, often indicating a T-cell exhaustion phenotype (23). The differences between the role of these 2 pathways on T-regulatory cells is of particular interest, as regulatory T cells also interact with a diverse range of resident cells in the central nervous system, resulting in a powerful neuroprotective effect in neuronal diseases, and therefore act as a critical regulator of immune homeostasis along the gut-brain axis (31). Studying the pathogenesis of persistent GI symptoms after ICI therapy may provide further insights into the role of the CTLA-4 pathway in enteric nerve function more broadly.

While colon inflammation is the most common mucosal site of inflammation in patients with GI-irAEs, a proportion may also experience concomitant or isolated small intestinal or upper GI inflammation (32,33). Similarly, in our study, most patients developed ICI colitis and about one-fourth experienced enteritis and/or gastritis. With the most common residual symptom being constipation, the presence of foregut involvement may reflect concepts related to central sensitization, in which inflammation on any spectrum of GI tract may then lead to downstream enteric neuron consequences with varying phenotypic symptoms. Several existing studies support central sensitization as an important pathophysiological mechanism in IBS and IBD. Notably, repetitive inflammatory episodes in IBD may result in prolonged hypersensitivity of peripheral nociceptors and in higher pain processing areas of the spinal cord and/or supraspinal nervous system, which may persist after inflammation has resolved (34,35). These changes may result in central sensitization, considered a potential pathophysiologic factor in DGBI (36).

While CTCAE is an effective clinical tool to classify patient symptoms and for following adverse events after ICI therapy, the score does not correlate with endoscopic mucosal severity measures in GI-irAE nor does it predict the response to enterocolitis therapy (10,21). Similarly, we found that neither severity of symptoms based on CTCAE score nor endoscopic severity of inflammation was predictive of developing persistent GI symptoms. This is concordant with the presentation and clinical course of postinfection IBS, as there are little data to support that severity of the initial enteric infection is associated with the risk of developing postinfection IBS (37). Similarly, underlying severity of IBD and the subsequent development of IBS/IBD overlap are not necessarily associated (17).

GI-irAE may be managed with anti-TNFα therapy without impacting tumor response and without dose-limiting toxic effects (38,39). In our study, a greater proportion of those who received anti-TNFα therapy did not develop persistent GI symptoms relative to those who did not undergo anti-TNFα therapy, suggesting that TNFα blockade may modify this risk. TNFα is a proinflammatory cytokine that plays an important role in the regulation of intestinal homeostasis (40). In ICI colitis, an inflammatory macrophage population responds to and produces TNFα and other chemokines, which likely results in the recruitment of additional T cells from the blood into the colon, further exacerbating colitis (24,41). TNFα release can also cause centrally mediated hypersensitization of the enteric nervous system (42,43). This may lower the threshold for visceral pain and may affect distension-induced contractions of the intestine. Attenuating the TNFα pathway in the treatment of GI-irAE may modulate the risk of developing persistent GI symptoms. The molecular basis of this risk modification should be further studied, which may provide a better understanding of the pathogenesis of postinflammatory DGBI.

The development of ICIs has heralded a new era in cancer treatment, enabling the long-term survival in patients with metastatic disease. As such, characterizing the long-term implications of undergoing ICI therapy has grown in importance. An abundance of evidence exists describing the acute clinical toxicities of these agents in the GI tract, although chronic effects are not yet entirely understood. There is some literature describing the longer-term sequelae of ICI therapy in those with endocrinologic, neurologic, and rheumatologic toxicities, although none in the GI tract to our knowledge. Chronic GI symptoms are known to be highly prevalent, contribute significantly to healthcare expenditures, as well as lead to poor quality of life and psychosocial outcomes (44,45). Such adverse outcomes disproportionately impair cancer survivors. Furthermore, we know that patients with cancer commonly experience GI symptoms while undergoing treatment for malignancy (e.g., while on chemotherapy, targeted therapy, or on opiates); however, rates of GI symptom development after achieving remission are unknown. Our data have important clinical implications and provides innovative considerations in the long-term management of cancer survivors who have experienced a GI-irAE and develop persistent GI symptoms.

This is the first study to explicitly describe the development of persistent GI symptoms after GI-irAE. We include a relatively homogenous population of patients with melanoma managed at a high-volume cancer center with expertise in the management of GI-irAE, allowing the benefit of retrospectively following these patients over a long period of time. We were able to include detailed data on the history and management of patients' malignancy and GI-irAE, which provides a greater understanding of our underlying aims. The primary exposure (GI-irAE) was rigorously adjudicated with clinical, endoscopic, and histologic evidence.

Limitations include that it was single center and retrospective in design, as well as the small sample size which limits our ability to make additional comparisons (type II error). Furthermore, the relatively high mortality rate of these patients may lead to immortal time bias. This study design is also subject to underreporting of GI symptoms by patients and underdocumentation of chronic GI symptoms by providers. For example, while the most common residual symptom was constipation, information about other associated symptoms is limited, and therefore, whether constipation is driven by a change in perception due to sensitizing effects from intestinal inflammation or dysmotility from intrinsic mechanisms is unclear. We also did not exclude patients on over the counter laxatives or fiber supplementation if they did not have an associated ICD9 or 10 code for a Rome IV diagnosis (46). Owing to limitations of retrospective chart review, we were unable to rule out underlying ongoing inflammatory activity as the cause for persistent GI symptoms, since only a subset of patients had repeat endoscopy after treatment of GI-irAE. We were also unable to ascertain Rome IV diagnoses that developed after GI-irAE, although hypothesize that long-term GI symptoms may serve as a surrogate for those in whom such diagnoses may be considered. With our cohort, it is also impossible to say definitively whether new GI symptoms were related to ICI therapy or another concurrent process, including the psychosocial trauma of a cancer diagnosis and treatment; subsequent comparisons with long-term ICI therapy survivors who did not develop ICI enterocolitis will be necessary to address this question. Last, we did not collect patient samples for the great majority of these patients and cannot report on microbiome-related or immunologic data.

In summary, 27% of patients with melanoma who underwent ICI therapy complicated by GI-irAE in this cohort developed persistent GI symptoms, with the most common symptom being constipation. Treatment with CTLA-4 inhibitors was independently associated with increased risk for developing persistent GI symptoms. A more comprehensive understanding of the relationship between GI-irAE and persistent GI symptoms may inform the nexus between the mechanosensory and immunologic phenomena underlying DGBI, and it may help improve the long-term management of cancer survivors.

## CONFLICTS OF INTEREST

**Guarantor of the article:** Michael Dougan, MD, PhD.

**Specific author contributions:** S.V., K.S., M.D. were involved in study design, analysis of data, interpretation of data, and drafting and writing of the manuscript. All authors were involved in collection and analysis of data and reviewing of the manuscript. All authors made significant contributions to the manuscript including drafting and revision of the article, and approval of the submission.

**Financial support:** K.S. is supported by NIH K23 DK120945.

**Potential competing interests:** K.S. has served as a consultant to Anji, Ardelyx, GI Supply, Mahana, Restalsis, and Sanofi.

**Study Highlights**

**WHAT IS KNOWN**

✓ Disorders of gut-brain interaction are highly prevalent and may develop in those with inflammatory conditions such as inflammatory bowel disease or after infectious gastroenteritis.
✓ The incidence and distribution of persistent gastrointestinal (GI) symptoms after immune checkpoint inhibitor-associated gastrointestinal toxicities are poorly documented.

**WHAT IS NEW HERE**

✓ About one quarter of patients with melanoma who underwent treatment with an immune checkpoint inhibitor and experienced a GI toxicity developed persistent GI symptoms.
✓ The most common symptom was constipation, followed by bloating and diarrhea.
✓ Use of a cytotoxic T lymphocyte-associated protein-4 specific immune checkpoint inhibitor was associated with development of persistent GI symptoms after immune checkpoint inhibitor GI toxicity.
